# A PILOT STUDY OF A PEER MENTORING SUPPORT INTERVENTION FOR CHINESE AMERICAN DEMENTIA CAREGIVERS

**DOI:** 10.1093/geroni/igad104.1088

**Published:** 2023-12-21

**Authors:** Jinyu Liu, Ethan Siu Leung Cheung, Yifan Lou

**Affiliations:** Baylor University, Waco, Texas, United States; University of Utah, Salt Lake City, Utah, United States; Yale University, New Haven, Connecticut, United States

## Abstract

**Objectives:**

Although many older Chinese Americans are expected to need intensive care because of cognitive impairment, a large gap exists in development of culturally sensitive interventions to reduce stress among caregivers in Chinese American communities. Informed by the sociocultural stress and coping model, we developed and tested a peer mentoring program. In the intervention, experienced Chinese caregivers are recruited and trained to be volunteering mentors, who provide three-month mentoring support to newer caregivers in the same ethnic community.

**Methods:**

A pilot randomized controlled trial was conducted among 38 Chinese American caregivers in New York City. During the three-month intervention, the 19 caregivers in the intervention group were paired with and weekly supported by 8 mentors via telephone calls. The control group received educational materials on caregiving skills and self-care. Three outcome variables, caregiving competence, loneliness, caregiver burden and depressive symptoms, were measured at the baseline, the 3-month follow-up and the 6-month follow-up.

**Results:**

T-test results show no significant differences in any outcome measures at the baseline between the two groups. Compared to the control group, the intervention group had larger reduced scores in loneliness at the 3-month follow-up (p=0.047) and caregiver burden (p=0.057) and depressive symptoms at the 6-month follow-up (p=0.057). We did not identify any significant differences in caregiving competence changes identified between the intervention and control groups.

**Conclusion:**

The pilot results indicate the potential effectiveness of empowering existing human resources of experienced caregivers in the same ethnic community in reducing stress of Chinese American dementia caregivers.

